# Enhancing Older Adults’ Lives Through Positive Aging Perception, Quality-of-Life Enhancement, and Social Support to Drive Acceptance and Readiness Toward Indoor Assistive Technology: Cross-Sectional Study

**DOI:** 10.2196/59665

**Published:** 2025-02-05

**Authors:** Ka Po Wong, Pei-Lee Teh, Weng Marc Lim, Shaun Wen Huey Lee

**Affiliations:** 1 Department of Applied Social Sciences The Hong Kong Polytechnic University Hong Kong China (Hong Kong); 2 Gerontechnology Laboratory and School of Business Monash University Malaysia Sunway City Malaysia; 3 Sunway Business School Sunway University Sunway City Malaysia; 4 School of Pharmacy Monash University Malaysia Sunway City Malaysia

**Keywords:** indoor assistive technology, positive aging perceptions, quality of life, social support, technology acceptance, technology readiness

## Abstract

**Background:**

The growing aging population faces increasing mobility limitations, highlighting the need for assistive technologies as potential solutions. These technologies support the independence and well-being of older adults and individuals with mobility challenges. Indoor mobility is essential for daily activities and significantly impacts their lives. Limited indoor mobility can reduce quality of life and heighten the risk of falls.

**Objective:**

This study explores how positive aging perceptions, quality-of-life enhancements, and social support influence the acceptance and readiness of indoor assistive technologies among older adults.

**Methods:**

A cross-sectional study was conducted at a gerontechnology laboratory, requiring participants to visit the facility in person. Each 60-minute session included demonstrations of various indoor assistive technologies and the completion of a questionnaire. The assistive technologies showcased encompassed a wide range of devices. Participants’ positive aging perceptions, quality-of-life enhancements, social support, technology acceptance, and readiness were measured using validated scales. Data were analyzed with AMOS (version 28; IBM Corp) and SPSS (version 28; IBM Corp), using structural equation modeling and multivariate analysis of covariance to assess the effects of predictors while controlling for demographic factors.

**Results:**

A total of 104 older adults aged 60 years and older participated, with a mean age of 67.92 (SD 5.68) years. Structural equation modeling indicated that positive aging perception has a significant influence on older adults’ control beliefs (*P*=.095), comfort (*P*=.047), and confidence (*P*<.001) in gerontechnology. Multivariate analysis revealed significant combined effects of quality-of-life enhancement (*P*=.01) and social support (*P*=.03) on technology acceptance and readiness, wherein quality-of-life enhancement (*P*=.001) and social support (*P*=.008) negatively impacted security perception. Among demographic variables, educational level significantly impacted gerontechnology confidence (*P*=.004) while ethnicity influenced optimism (*P*=.003).

**Conclusions:**

This study sheds light on key factors affecting older adults’ acceptance and readiness to adopt indoor assistive technologies. Findings highlight the importance of fostering positive aging perceptions through these technologies. Addressing issues related to control beliefs, comfort, and confidence in gerontechnology is essential to enhance technology acceptance and readiness among older adults. Future research should investigate the underlying mechanisms and create targeted interventions to support successful technology adoption in this population.

## Introduction

### Background

Recent years have seen an increased focus on addressing the needs of older adults and individuals with mobility challenges [1]. As the global population ages, the prevalence of mobility challenges has become a significant societal concern [2]. The World Health Organization (WHO) projects that by 2050, the number of people aged 60 years and older will reach 2 billion, with a substantial portion experiencing mobility limitations [3]. This demographic shift has intensified efforts to develop innovative solutions that support the independence and well-being of older adults and those with mobility challenges [4].

Indoor mobility, which includes activities such as sitting, standing, and walking, is essential for maintaining independence and overall well-being, especially among older adults and individuals with mobility difficulties [5,6]. Limited indoor mobility can lead to serious consequences, including an increased risk of falls and a diminished quality of life [5]. Therefore, enhancing indoor mobility is crucial for enabling these individuals to participate actively in daily activities and sustain their independence [7]. Assistive technologies, such as commodes, home care beds, and reclining wheelchairs, have emerged as solutions to address the specific needs of this population. These technologies help overcome mobility limitations and improve functional abilities [8], potentially transforming how older adults and individuals with mobility challenges interact with their living environments. Successful integration of assistive technologies requires careful selection and personalization to match the unique capabilities, needs, and preferences of each user [9].

Indoor assistive technologies encompass a wide range of tools, devices, and equipment designed to enhance and support the independence and mobility of older adults and individuals with functional limitations within indoor settings [10]. These technologies include mobility aids (eg, scooters, walkers, and wheelchairs), transfer and positioning devices (eg, grab bars, patient lifts, and reclining chairs), smart home systems (eg, automated lighting and voice-controlled appliances), and various adaptive equipment (eg, bed rails, shower chairs, and toilet risers). Tailored to address the specific challenges faced by older adults and those with mobility challenges, these technologies facilitate daily activities and help maintain independence at home and in other indoor environments [11]. Through targeted support to increase functional capacity, indoor assistive technologies empower older adults and individuals with mobility challenges, enhancing their daily lives [12] and improving overall quality of life and well-being [13,14].

Gerontechnology, the intersection of gerontology and advanced technology, aims to enhance the health, independence, and quality of life of older adults [15]. Incorporating gerontechnological advancements into indoor assistive technologies addresses the unique challenges faced by this population, fostering greater independence and well-being [15]. Examples of gerontechnology applications include advanced mobility aids such as smart walkers and wheelchairs, cognitive support tools such as memory aids and smart home systems, and social engagement platforms such as web-based communication tools [16]. These technologies assist older adults in maintaining physical movement, performing daily activities, and reducing social isolation, thereby creating an environment where they can live independently and with dignity. Acceptance and readiness to adopt these technologies are influenced by positive aging perceptions, enhanced quality of life, and social support systems. This study examines how these factors drive technology acceptance and readiness among older adults, ultimately improving their lives through indoor assistive technologies.

### Theoretical Background

This study is grounded in the Theory of Planned Behavior (TPB) [17]. The TPB posits that individuals’ behavioral intentions are influenced by 3 primary constructs: attitudes, subjective norms, and perceived behavioral control. In the context of this study, attitudes represent older adults’ overall evaluation and perception of indoor assistive technologies. This study investigates whether older adults who hold a positive aging perception and believe that assistive technologies enhance their quality of life are more likely to adopt and use these technologies [18,19].

Subjective norms within the TPB refer to the social influences and societal expectations that shape an individual’s behavior [20]. In this study, subjective norms are assessed through the role of social support—specifically, the assistance provided by family and friends—in influencing the acceptance and readiness to adopt indoor assistive technologies among older adults.

Perceived behavioral control is another critical construct of the TPB, encompassing individuals’ beliefs and perceptions about their ability to perform a specific behavior [21,22]. This study measures perceived behavioral control by evaluating older adults’ perceptions of their confidence and readiness to adopt and use indoor assistive technologies.

**Figure 1:**
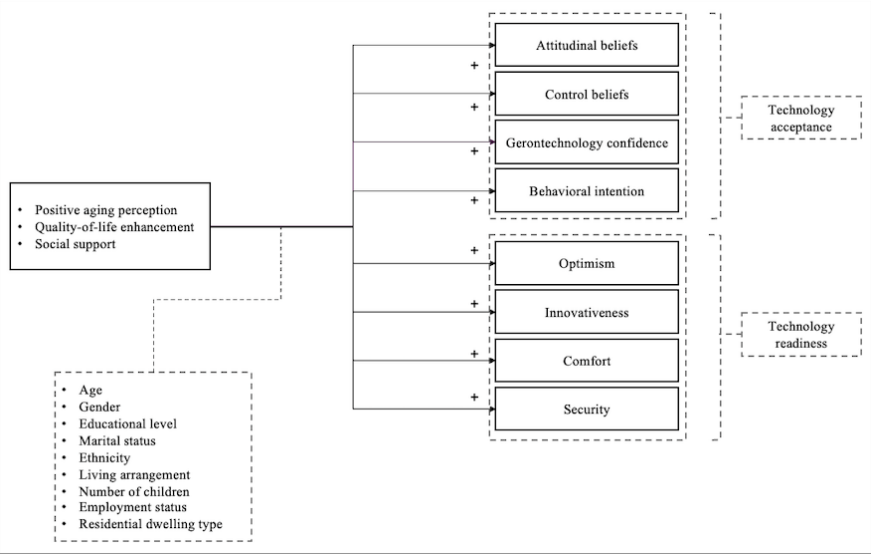
Study conceptual model.

Applying the TPB enables this study to identify the factors that drive various aspects of older adults’ acceptance and readiness to embrace indoor assistive technologies. Such insights are valuable for developing interventions and strategies aimed at promoting engagement, well-being, and overall quality of life among older adults [23]. The study’s conceptual model is illustrated in Figure 1.

Recognizing the needs of older adults with diminished mobility highlights the importance of creating innovative strategies to enhance their independence and physical and psychological well-being [1]. Although existing studies have examined the perceptions of technologies among older adults with and without disabilities or impairments, there remains a need for more evidence-based research focused on the specific factors that influence the acceptance and readiness to adopt indoor assistive technologies. Understanding these factors can provide promising pathways for improving indoor mobility and enhancing the quality of life for this population [13,22].

Previous research has explored various dimensions of indoor assistive technologies. For example, Gitlin et al [24] discovered that assistive devices and home modifications significantly improved functional abilities and reduced the risk of falls among older adults, indicating positive attitudes toward these technologies. Similarly, several studies have shown that the use of assistive devices in home environments leads to increased independence and reduced caregiver burden [15,25], underscoring the role of social support in technology acceptance. In addition, Demiris et al [26] and Liu et al [27] examined older adults’ perceptions of their ability to use smart home technologies, aligning with the concept of perceived behavioral control in the TPB [17]. More recent studies, such as Peek et al [19], have identified key factors influencing the acceptance of technology for aging in place, including perceived ease of use, usefulness, and social influence, while Mitzner et al [28] highlighted the importance of user experience in technology acceptance among older adults. These studies provide empirical evidence supporting the benefits of indoor assistive technologies and emphasize the necessity of understanding user acceptance and readiness.

The global demographic shift toward an aging population and the rising prevalence of mobility limitations present urgent challenges that must be addressed. With the projected increase in the number of older adults, developing interventions and strategies that effectively cater to their unique needs and preferences becomes crucial [4]. To achieve a comprehensive understanding of older adults’ needs regarding assistive technologies, targeted strategies must investigate their perspectives, acceptance, and readiness of aging in relation to indoor assistive technologies. Conducting such research can bridge existing knowledge gaps and inform the creation of strategies tailored to the specific needs of older adults, ultimately enhancing their overall independence and well-being [5].

This study aims to explore the influences of positive aging perception, quality-of-life enhancement through assistive technologies, and social support on technology acceptance and readiness among older adults in an indoor setting. Specifically, this study seeks to understand how these factors interact and contribute to older adults’ attitudes and readiness to adopt and use indoor assistive technologies. The hypothesis posits that a positive aging perception, improved quality of life, and supportive social relationships positively affect older adults’ technology acceptance, including attitudinal beliefs, control beliefs, confidence in gerontechnology, and behavioral intention. It is further hypothesized that these factors also positively impact older adults’ readiness, which encompasses comfort, innovativeness, optimism, and security.

## Methods

### Study Setting

This study used a cross-sectional experiential design conducted at a gerontechnology laboratory located at an Australian university’s international branch campus in Malaysia. Participants were required to visit the laboratory in person to take part in the study. Each session lasted approximately 60 minutes and included active participation in demonstrations of various indoor assistive technologies, followed by the completion of a questionnaire. The assistive technology demonstrations encompassed a wide range of devices tailored to support mobility.

### Participants Recruitment

Recruitment efforts targeted individuals through advertisements placed across multiple social media platforms and in universities. Interested participants registered their interest by completing an online registration form through Google Forms or by contacting the provided phone number. Eligibility criteria were established to ensure suitable participation, including being aged 60 years or older; not having severe mobility challenges that would prevent full engagement in the experiential session; the ability to attend the demonstration session; and the capacity to provide written informed consent. These criteria ensured that participants could actively engage in the study activities and provide meaningful feedback on the assistive technologies demonstrated.

### Study Design

**Figure 2:**
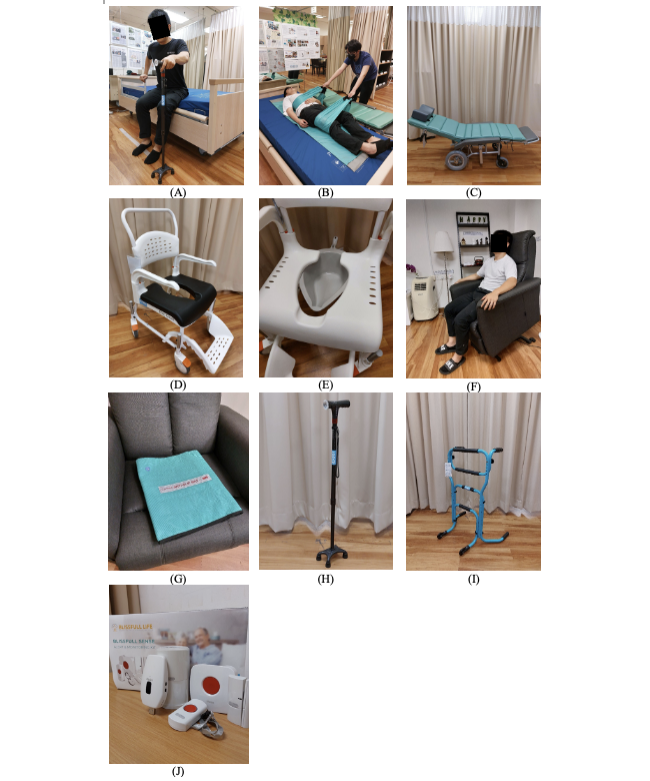
Assistive technologies demonstrated in the experiential session: (A) handling side guard and home care bed; (B) transfer roller kit board; (C) tilt and reclining wheelchair; (D) shower commode chair; (E) commode with mobile pan; (F) recliner and stand-up sofa; (G) antislip seating mat; (H) carbon fiber quad cane; (I) 3-in-1 stand assist walker; and (J) nonintrusive activity-based sensor monitoring system.

Participants engaged in an experiential session featuring various indoor assistive technologies (given in Figure 2A-J). Each demonstrated device is specifically designed to support older adults and individuals with mobility challenges within their living environments [29]. The session included the following technologies: The SafeFree handling side guard and home care bed (Figure 2A) provide safe and comfortable support for resting, sleeping, and transferring activities within the home. The transfer roller kit board (Figure 2B) assists with safe transfers from beds, chairs, or the floor, enhancing indoor mobility. The tilt and reclining wheelchair (Figure 2C) allows users to adjust their position and posture to perform different activities at home. The shower commode chair (Figure 2D) enables individuals to bathe and use the toilet safely within their living spaces. The commode with a mobile pan (Figure 2E) offers discreet and convenient toileting support indoors. The recliner and stand-up sofa (Figure 2F) incorporate features that aid in sitting, standing, and repositioning for those with mobility challenges. The antislip seating mat (Figure 2G) enhances stability and safety on various seating surfaces within the home. The carbon fiber quad cane (Figure 2H) provides additional support and stability for individuals with limited mobility. The 3-in-1 stand assist walker (Figure 2I) supports users in performing various daily activities safely indoors. Finally, the nonintrusive activity-based sensor monitoring system (Figure 2J) offers remote monitoring and support for individuals in their living spaces.

A trained research assistant conducted the demonstrations to ensure consistency and quality throughout the session. Each participant received personalized attention from a single research assistant to maintain fidelity. The research team reviewed and approved all demonstration materials to ensure accuracy and effectiveness. Before the study, the research assistant conducted a trial demonstration for the research team, evaluating the clarity and conveyance of the content. In addition, a research team member conducted random visits to demonstration sessions to verify that the content remained consistent and adhered to the predetermined standards.

### Outcome Measures

Participants were assessed after the demonstration session using a questionnaire divided into 5 main sections: positive aging perception, quality-of-life enhancement, social support, technology acceptance, and technology readiness.

#### Positive Aging Perception

The Awareness of Age-Related Change (AARC) questionnaire [30] was used to assess participants’ positive aging perception. A total of 10 items were selected for this study, using a 5-point Likert scale ranging from 1 (not at all) to 5 (very much). The internal consistency reliability demonstrated strong results for both AARC-Gains (α=.82) and AARC-Losses (α=.78) [31].

#### Quality-of-Life Enhancement

Quality-of-life enhancement was measured based on Moxley et al [32]. This section comprised 5 items evaluated using a 7-point Likert scale, from 1 (Not at all) to 7 (A lot). The items specifically addressed the extent to which assistive technologies can improve the quality of life for older adults. This scale has been widely adopted in gerontechnology research including studies by Dale et al [33].

#### Social Support

Social support was assessed using a 5-item scale adapted from Moxley et al [32]. This scale evaluated the level of assistance required from family and friends in becoming proficient with the presented technologies. Responses were measured on a 7-point Likert scale, ranging from 1 (Not at all) to 7 (A lot). The scale’s widespread use in gerontechnology research, as demonstrated by its application in Dale et al [33], underscores its validity and reliability.

#### Technology Acceptance

Technology acceptance was evaluated using the Senior Technology Acceptance Model questionnaire, adapted from Chen and Lou [34] and Venkatesh et al [35]. This instrument included 4 variables: attitudinal beliefs (3 items), control beliefs (4 items), gerontechnology confidence (gerontechnology anxiety; 2 items), and behavioral intention (3 items). Participants responded on a 7-point Likert scale, from 1 (Strongly disagree) to 7 (Strongly agree). The questionnaire demonstrated strong internal consistency, with α values ranging from 0.96 to 0.97 [36]. Its widespread adoption across various fields highlights its effectiveness in assessing users’ perceptions and attitudes toward technology adoption and utilization [37].

#### Technology Readiness

The Technology Readiness Questionnaire (TRQ) developed by Parasuraman and Colby [38] was used to measure participants’ readiness and willingness to embrace technology. The TRQ includes 4 dimensions: comfort, innovativeness, optimism, and security, with 4 items assigned to each dimension. Participants rated each item on a 5-point Likert scale, where higher scores indicated a greater inclination to adopt and use technology. The TRQ’s extensive application in aging-related research emphasizes its relevance in evaluating individuals’ attitudes and preparedness for technology adoption [39].

### Analytical Techniques

The measurement model was validated using confirmatory factor analysis (CFA) with SPSS (version 28.0; IBM Corp) and AMOS (version 28; IBM Corp). CFA evaluated the validity of the measurement model by examining the relationships between observed variables and their underlying latent constructs. A total of 8 key goodness-of-fit indices, recommended by Hu and Bentler [40], were used to assess the model fit. These indices included Cronbach α, chi-square and its respective degrees of freedom (*χ*²/*df*), goodness-of-fit index (GFI), incremental fit index (IFI), comparative fit index (CFI), Tucker-Lewis index (TLI), root mean square error of approximation (RMSEA), and parsimony goodness-of-fit index (PGFI).

Descriptive statistics summarized the demographic variables, providing the mean and SD for each measure to offer an overview of the sample characteristics. Correlation analysis was performed to provide a preliminary assessment of the relationships between variables, while structural equation modeling explored the main relationships between predictor and outcome variables. Multivariate analysis of covariance (MANCOVA) was used as a post hoc analysis to scrutinize the effects of predictor variables on outcome variables while controlling for demographic characteristics such as age, gender, education level, and ethnicity. Wilks lambda (λ) was used to assess multivariate effects, followed by univariate analyses to identify specific between-subjects effects. Effect sizes were reported using partial eta squared (η²). Statistical significance was set at *P*<.05, with marginal significance noted at *P*<.10.

### Ethical Considerations

The research protocol was developed by Monash University Malaysia, and study approval was granted by the Monash University Human Research Ethics Committee (project ID: 39857; review reference: 2023-39857-98651) in September 2023. All participants provided written informed consent and received a token of appreciation of RM 50 (approximately US $11.2) for their participation. To ensure participant confidentiality, all collected data will be anonymized. In instances where full anonymization is not possible, strict protective measures will be implemented, including secure data storage and restricted access to authorized personnel only, to safeguard participant information.

## Results

### Participant Characteristics

A total of 104 older adults participated in the study, with an average mean age of 67.92 (SD 5.68) years. The sample comprised 58.7% (61/104) women and 41.3% (43/104) men. Educational levels varied, including 2.9% (3/104) with primary education and below, 24% (25/104) with secondary education, 27.9% (29/104) holding a diploma or preuniversity qualification, 29.8% (31/104) possessing a degree or professional certification, and 15.4% (16/104) with postgraduate degrees. Most participants were married (73/104, 70.2%), followed by those who were never married (5.8%, 6/104), divorced (9/104, 8.7%), widowed or widowers (10/104, 9.6%), separated (5/104, 4.8%), and others (1/104, 1%). Ethnically, the majority identified as Chinese (86/104, 82.7%), with smaller representations of Indian (16/104, 15.4%), Malay (1/104, 1%), and other ethnicities (1/104, 1%). Regarding living arrangements, 78.8% (82/104) resided with household members, 17.3% (18/104) lived alone, and 3.8% (4/104) had other living situations. Participants reported having varying numbers of children, with 14.4% (15/104) having none, 14.4% (15/104) having 1 child, 32.7% (34/104) having 2 children, 32.7% (34/104) having 3 children, 1.9% (2/104) having 4 children, and 2.9% (3/104) having 5 children. In terms of employment status, 57.7% (60/104) were private retirees, 11.5% (12/104) were government retirees (including pensioners), 8.7% (9/104) were self-employed, 5.8% (6/104) were private sector employees, 7.7% (8/104) were homemakers, 5.8% (6/104) were unemployed, and 2.9% (3/104) were in other categories. The majority of participants lived in terraced, linked houses, or semidetached homes (60/104, 57.7%), followed by flats, apartments, condominiums, or townhouses (34/104, 32.7%); detached houses (9/104, 8.7%); and other dwelling types (1/104, 1%). Statistical analysis revealed that living arrangements significantly varied within the sample (*P*=.002), while other demographic variables did not show significant differences (Table 1).

**Table 1:** Demographic information (N=104).

Demographics		Values		*P* value
**Age (years), mean (SD)**		67.92 (5.68)		.22
**Gender, n (%)**				.08
	Men	43 (41.3)		
	Women	61 (58.7)		
**Educational level, n (%)**				.97
	Primary school and below	3 (2.9)		
	Secondary school	25 (24)		
	Diploma or preuniversity	29 (27.9)		
	Degree or professional	31 (29.8)		
	Postgraduate	16 (15.4)		
**Marital status, n (%)**				.16
	Never married	6 (5.8)		
	Married	73 (70.2)		
	Separated	5 (4.8)		
	Divorcee	9 (8.7)		
	Widow or widower	10 (9.6)		
	Others	1 (1)		
**Ethnicity, n (%)**				.18
	Malay	1(1)		
	Chinese	86 (82.7)		
	Indian	16 (15.4)		
	Others	1 (1)		
**Living arrangement, n (%)**				.002
	Living alone	18 (17.3)		
	Living with household members	82 (78.8)		
	Others	4 (3.8)		
**Number of children, n (%)**				.39
	0	15 (14.4)		
	1	15 (14.4)		
	2	34 (32.7)		
	3	34 (32.7)		
	4	2 (1.9)		
	5	3 (2.9)		
**Employment status, n (%)**				.41
	Private sector employee	6 (5.8)		
	Self-employed	9 (8.7)		
	Government retirees (including pensioners)	12 (11.5)		
	Private retiree	60 (57.7)		
	Homemaker	8 (7.7)		
	Unemployed	6 (5.8)		
	Others	3 (2.9)		
**Residential dwelling type, n (%)**				.18
	Flat, apartment, condominium, or townhouse	34 (32.7)		
	Detached house (bungalow or traditional house)	9 (8.7)		
	Terrace, link house, or semidetached	60 (57.7)		
	Others	1 (1)		

### Correlations

The correlation analysis in Table 2 identified several significant relationships among the study variables. Age positively correlated with marital status (*r*= 0.278; *P*=.004), indicating that older participants were more likely to be married. Gender exhibited a significant negative correlation with living arrangements (*r*=–0.309; *P*=.001), suggesting that women were more likely to live with household members compared with men. Educational level was negatively associated with employment status (*r*=–0.233; *P*=.02). Residential dwelling type showed a positive correlation with living arrangements (*r*=0.397; *P*<.001) and the number of children (*r*=0.207; *P*=.04), indicating that certain housing types were more common among those living with more children. Quality-of-life enhancement was negatively related to age (*r*=–0.220; *P*=.02) and educational level (*r*=–0.201; *P*=.04). Social support positively correlated with quality-of-life enhancement (*r*=0.223; *P*=.02) and negatively correlated with gerontechnology confidence (*r*=–0.317; *P*=.001). Behavioral intention was positively associated with quality-of-life enhancement (*r*=0.246; *P*=.01), attitudinal beliefs (*r*=0.275; *P*=.005), control beliefs (*r*=0.396; *P*<.001), gerontechnology confidence (*r*=0.206; *P*=.04), optimism (*r*=0.428; *P*<.001), and innovativeness (*r*=0.204; *P*=.04). These correlations offer preliminary insights on how demographic factors and positive perceptions of aging, quality of life, and social support influence technology acceptance and readiness among older adults.

**Table 2:** Correlation matrix.

	1	2	3	4	5	6	7	8	9	10	11	12	13	14	15	16	17	18	19	20
**1. Age (mean 67.92, SD 5.68)**																				
*r*	1	–.063	–.131	.278	.112	–.135	.199	–.014	.019	–.020	–.220	.01	–.129	.195	.042	–.068	.063	.132	.089	–.034
*P* value	—^a^	.52	.18	.004	.26	.17	.04	.89	.84	.84	.02	.92	.19	.047	.67	.49	.52	.18	.37	.73
**2. Gender^b^ (mean 1.59, SD 0.495)**																				
*r*	–.063	1	.003	.14	.131	–.309	–.085	.08	–.133	.085	–.143	.096	.025	.035	–.045	–.010	–.050	–.189	–.099	–.150
*P* value	.52	—	.97	.16	.18	.001	.39	.42	.18	.39	.15	.33	.80	.72	.65	.92	.62	.06	.32	.13
**3. Educational level^c^ (mean 3.31, SD 1.089)**																				
*r*	–.131	.003	1	–.162	–.140	–.045	–.137	–.233	.059	.178	–.201	–.289	.125	.114	.406	.088	.011	.025	.051	.095
*P* value	.18	.97	—	.101	.16	.65	.17	.02	.55	.07	.04	.003	.21	.25	<.001	.38	.91	.80	.61	.34
**4. Marital status^d^ (mean 2.49, SD 1.115)**																				
*r*	.278	.14	–0.162	1	.042	–.089	.107	.117	.007	.159	–.034	.025	.103	.169	.025	.008	–.045	.277	–.002	.048
*P* value	.004	.16	.101	—	.67	.37	.28	.24	.95	.11	.73	.80	.30	.09	.80	.94	.65	.004	.98	.63
**5. Ethnicity^e^ (mean 2.19, SD 0.609)**																				
*r*	.112	.131	–.140	.042	1	.125	.046	.019	.128	–.043	.269	.260	.054	.146	–.030	.214	.268	.043	.098	–.092
*P* value	.26	.18	.16	.67	—	.21	.64	.85	.19	.66	.006	.008	.58	.14	.76	.03	.006	.66	.32	.36
**6. Living arrangement^f^ (mean 1.9, SD 0.566)**																				
*r*	–.135	–.309	–.045	–.089	.125	1	.129	–.002	.397	–.060	.079	–.078	.065	.02	.182	.07	.16	.024	.195	.244
*P* value	.17	.001	.65	.37	.21	—	.19	.99	<.001	.55	.42	.43	.51	.84	.06	.48	.11	.81	.047	.01
**7. Number of children (mean 2.02, SD 1.188)**																				
*r*	.199	–.085	–.137	.107	.046	.129	1	–.111	.207	.043	–.001	.026	–.135	.052	.004	.011	.085	.031	–.077	–.055
*P* value	.04	.39	.17	.28	.64	.19	—	.26	.04	.66	.99	.80	.17	.60	.97	.91	.39	.75	.44	.58
**8. Employment status^g^ (mean 5.67, SD 1.517)**																				
*r*	–.014	.08	–.233	.117	.019	–.002	–.111	1	.01	–.017	.049	.002	–.105	–.108	.01	–.062	–.075	.152	.067	.031
*P* value	.89	.42	.02	.24	.85	.99	.26	—	.92	.86	.62	.98	.29	.28	.92	.53	.45	.12	.50	.75
**9. Residential dwelling type^h^ (mean 2.28, SD 0.96)**																				
*r*	.019	–.133	.059	.007	.128	.397	.207	.01	1	–.016	–.010	–.162	–.064	–.088	.093	.036	.105	.034	.104	.179
*P* value	.84	.18	.55	.95	.19	<.001	.04	.92	—	.87	.92	.101	.52	.38	.35	.71	.29	.73	.29	.07
**10. Positive ageing perception (mean 3.959, SD 0.404)**																				
*r*	–.020	.085	.178	.159	–.043	–.060	.043	–.017	–.016	1	–.077	–.090	.192	.053	.202	–.082	–.008	.073	.094	–.008
*P* value	.84	.39	.07	.11	.66	.55	.66	.86	.87	—	.44	.36	.051	.59	.04	.41	.93	.46	.34	.94
**11. Quality-of-life enhancement (mean 4.687, SD 1.356)**																				
*r*	–.220	–.143	–.201	–.034	.269	.079	–.001	.049	–.010	–.077	1	.223	.082	.175	–.061	.246	.201	.161	.072	.19
*P* value	.02	.15	.04	.73	.006	.42	.99	.62	.92	.44	—	.02	.41	.07	.54	.01	.04	.10	.47	.054
**12. Social support (mean 3.483, SD 1.295)**																				
*r*	.01	.096	–.289	.025	.260	–.078	.026	.002	–.162	–.090	.223	1	.16	.041	–.317	–.093	–.123	–.109	–.150	–.232
*P* value	.92	.33	.003	.80	.008	.43	.80	.98	.101	.36	.02	—	.104	.68	.001	.35	.21	.27	.13	.02
**13. Attitudinal beliefs (mean 5.401, SD 1.293)**																				
*r*	–.129	.025	.125	.103	.054	.065	–.135	–.105	–.064	.192	.082	.16	1	.276	.177	.275	.281	.129	.102	.152
*P* value	.19	.80	.21	.30	.58	.51	.17	.29	.52	.051	.41	.104	—	.005	.07	.005	.004	.19	.30	.12
**14. Control beliefs (mean 5.337, SD 0.931)**																				
*r*	.195	.035	.114	.169	.146	.02	.052	–.108	–.088	.053	.175	.041	.276	1	.201	.396	.177	.341	.022	.1
*P* value	.047	.72	.25	.09	.14	.84	.60	.28	.38	.59	.08	.68	.005	—	.04	<.001	.07	<.001	.82	.31
**15. Gerontechnology confidence (mean 4.88, SD 1.471)**																				
*r*	.042	–.045	.406	.025	–.030	.182	.004	.01	.093	.202	–.061	–.317	.177	.201	1	.206	.157	.003	.408	.335
*P* value	.67	.65	<.001	.80	.76	.06	.97	.92	.35	.04	.54	.001	.07	.04	—	.04	.11	.98	<.001	.001
**16. Behavioral intention (mean 5.804, SD 0.786)**																				
*r*	–.068	–.010	.088	.008	.214	.07	.011	–.062	.036	–.082	.246	–.093	.275	.396	.206	1	.428	.204	.076	.07
*P* value	.49	.92	.38	.94	.03	.48	.91	.53	.71	.41	.01	.35	.005	<.001	.04	—	<.001	.04	.44	.48
**17. Optimism (mean 4.276, SD 0.515)**																				
*r*	.063	–.050	.011	–.045	.268	.16	.085	–.075	.105	–.008	.201	–.123	.281	.177	.157	.428	1	.269	.145	.103
*P* value	.52	.62	.91	.65	.006	.11	.39	.45	.29	.93	.04	.21	.004	.07	.11	<.001	—	.006	.14	.30
**18. Innovativeness (mean 3.26, SD 0.637)**																				
*r*	.132	–.189	.025	.277	.043	.024	.031	.152	.034	.073	.161	–.109	.129	.341	.003	.204	.269	1	.173	.053
*P* value	.18	.06	.80	.004	.66	.81	.75	.12	.73	.46	.102	.27	.19	<.001	.97	.04	.006	—	.08	.59
**19. Comfort (mean 3.202, SD 0.626)**																				
*r*	.089	–.099	.051	–.002	.098	.195	–.077	.067	.104	.094	.072	–.150	.102	.022	.408	.076	.145	.173	1	.404
*P* value	.37	.32	.61	.98	.32	.047	.44	.50	.29	.34	.47	.13	.30	.82	<.001	.44	.14	.08	—	<.001
**20. Security (mean 2.752, SD 0.746)**																				
*r*	–.034	–.150	.095	.048	–.092	.244	–.055	.031	.179	–.008	.19	–.232	.152	.1	.335	.07	.103	.053	.404	1
*P* value	.73	.13	.34	.63	.36	.01	.58	.75	.07	.94	.054	.02	.12	.31	.001	.48	.30	.59	<.001	—

^a^Not applicable.

^b^Gender: 1=men and 2=women.

^c^Educational level: 1=primary school and below, 2=secondary school, 3=diploma or preuniversity, 4=degree or professional, and 5=postgraduate.

^d^Marital status: 1=never married, 2=married, 3=separated, 4=divorcee, 5=widow or widower, and 6=others.

^e^Ethnicity: 1=Malay, 2=Chinese, 3=Indian, 4=others.

^f^Living arrangement: 1=living alone, 2=living with household members, and 3=others.

^g^Employment status: 1=private sector employee, 2=self-employed, 3=government retiree (includes pensioners), 4=private retiree, 5=homemaker, 6=unemployed, and 7=others.

^h^Residential dwelling type: 1=flat, apartment, condominium, or townhouse; 2=detached house (bungalow or traditional house); 3=terrace, link house, or semidetached; 4=others.

### Measurement Model

The measurement model was validated via CFA. The Cronbach α values for positive aging perception, quality-of-life enhancement, social support, technology acceptance, and technology readiness were 0.736, 0.855, 0.815, 0.740, and 0.731, respectively, demonstrating acceptable internal consistency. Model fit indices were as follows: *χ*²_752_=1069.140, *P*<.001; GFI=0.703; IFI=0.858; CFI=0.850; TLI=0.829; RMSEA=0.064; and PGFI=0.585. These values met the recommended thresholds, indicating a moderately acceptable model fit [40].

### Structural Model

The structural model was tested via structural equation modeling (Table 3), illustrating how positive aging perception, quality-of-life enhancement, and social support relate to various aspects of technology acceptance and readiness among older adults. Positive aging perception significantly enhanced gerontechnology confidence (β=.462; *P*<.001) and comfort (β=.323; *P*=.047) and exhibited a marginally significant positive relationship with control beliefs (β=.228; *P*=.095). In contrast, quality-of-life enhancement and social support did not show significant effects on any of the technology acceptance or readiness components. These findings suggest that positive aging perception plays a crucial role in fostering confidence and comfort with gerontechnology, whereas quality-of-life enhancement and social support have limited impacts on technology acceptance and readiness within this population.

**Table 3:** Structural equation modeling results.

Path		Standardized coefficient (β)	Unstandardized coefficient (B)	SE	*t* value	*P* value
**Positive aging perception**						
	Attitudinal beliefs	.143	0.173	0.145	1.196	.23
	Control beliefs	.228	0.242	0.145	1.670	.095
	Gerontechnology confidence	.462	1.220	0.321	3.806	<.001
	Behavioral intention	.190	0.239	0.146	1.635	.102
	Optimism	.041	0.027	0.068	0.400	.69
	Innovativeness	.061	0.042	0.094	0.453	.65
	Comfort	.323	0.227	0.114	1.984	.047
	Security	.171	0.082	0.067	1.219	.22
**Quality-of-life enhancement**						
	Attitudinal beliefs	.097	0.932	1.687	0.552	.58
	Control beliefs	.031	0.262	1.012	0.259	.80
	Gerontechnology confidence	–.104	–2.177	3.615	–0.602	.55
	Behavioral intention	.266	2.652	3.973	0.667	.50
	Optimism	.165	0.878	1.421	0.618	.54
	Innovativeness	.149	0.829	1.382	0.600	.55
	Comfort	.160	0.896	1.475	0.607	.54
	Security	.150	0.572	0.953	0.600	.55
**Social support**						
	Attitudinal beliefs	.186	0.484	0.398	1.217	.22
	Control beliefs	.142	0.323	0.323	1.002	.32
	Gerontechnology confidence	–.215	–1.220	0.858	–1.422	.16
	Behavioral intention	–.124	–0.333	0.336	–.993	.32
	Optimism	–.192	–0.277	0.245	–1.130	.26
	Innovativeness	–.176	–0.264	0.242	–1.090	.28
	Comfort	–.289	–0.436	0.329	–1.323	.19
	Security	–.357	–0.367	0.279	–1.314	.19

### Post hoc Observations

The MANCOVA assessed the combined effects of independent variables and demographic factors on technology acceptance and readiness among older adults (Tables 4 and 5). The analysis revealed that positive aging perception did not significantly influence the various aspects of technology acceptance and readiness when controlling for demographic characteristics (Wilks λ=0.897, *F*_8,61_=0.877, *P*=.54, partial η²=0.103). This consistency reinforces the structural model findings, indicating that the relationships between positive aging perception and factors such as control beliefs, comfort, and gerontechnology confidence remain stable across different demographic groups. Conversely, quality-of-life enhancement (Wilks λ=0.737, *F*_8,61_=2.717, *P*=.01, partial η²=0.263) and social support (Wilks λ=0.762, *F*_8,61_=2.387, *P*=.03, partial η²=0.238) significantly impacted technology acceptance and readiness. A closer examination showed that both quality-of-life enhancement (*P*=.001) and social support (*P*=.008) negatively influenced security perceptions, suggesting that improvements in these areas are associated with reduced concerns about the security of assistive technologies. In addition, demographic factors played a notable role: educational level significantly predicted gerontechnology confidence (β=29.548, *P*=.004, partial η²=0.201) and ethnicity was a significant predictor of optimism (β=3.373, *P*=.003, partial η²=0.187). These findings indicate that while positive aging perceptions consistently affect certain aspects of technology acceptance, quality-of-life enhancements and social support are crucial in shaping specific perceptions such as security. Furthermore, educational attainment and ethnic background influence confidence and optimism regarding technology use. These granular insights highlight the importance of addressing both demographic and psychological factors to foster effective adoption and readiness for indoor assistive technologies among older adults. Further explanation is provided in the Discussion section.

**Table 4:** Multivariate analysis of covariance on the effects of predictor variables while controlling for demographic characteristics.

Variables		Wilks λ	*F* value (*df*)	*P* value	Partial η²
**Predictor variables**					
	Positive aging perception	0.897	0.877 (8, 61)	.54	0.103
	Quality-of-life enhancement	0.737	2.717 (8, 61)	.01	0.263
	Social support	0.762	2.387 (8, 61)	.03	0.238
**Demographic characteristics**					
	Age	0.770	1.066 (8, 61)	.39	0.123
	Gender	0.920	0.663 (8, 61)	.72	0.080
	Educational level	0.498	1.471 (8, 61)	.06	0.160
	Marital status	0.584	0.883 (8, 61)	.67	0.102
	Ethnicity	0.571	1.574 (8, 61)	.051	0.170
	Living arrangement	0.791	0.947 (8, 61)	.52	0.110
	Number of children	0.642	0.718 (8, 61)	.90	0.085
	Employment status	0.519	0.904 (8, 61)	.66	0.104
	Residential dwelling type	0.753	0.759 (8, 61)	.78	0.090
**Significant between-subjects path effects (** ***F*** **>3.00,** ***P*** **<.05)**					
	Quality-of-life enhancement: security	6.404	12.913 (1, 68)	.001	0.160
	Social support: security	3.674	7.408 (1, 68)	.008	0.098
	Educational level: gerontechnology confidence	29.548	4.266 (4, 68)	.004	0.201
	Ethnicity: optimism	3.373	5.198 (3, 68)	.003	0.187

**Table 5:** Multiple regression analyses examining the effects of predictor variables and demographic characteristics on technology acceptance and technology readiness outcomes.

Outcome variables		*R*²	Adjusted *R*²	*F* value (*df*)	*P* value
**Overall model effects** **(predictor variables+demographic characteristics=outcomes)**					
	Attitudinal beliefs	0.439	0.158	1.562 (7, 95)	.06
	Control beliefs	0.398	0.097	1.321 (7, 95)	.16
	Gerontechnology confidence	0.471	0.207	1.783 (7, 95)	.02
	Behavioral intention	0.303	–0.045	0.870 (7, 95)	.67
	Optimism	0.441	0.161	1.576 (7, 95)	.06
	Innovativeness	0.348	0.021	1.066 (7, 95)	.40
	Comfort	0.337	0.005	1.015 (7, 95)	.47
	Security	0.401	0.101	1.339 (7, 95)	.15

## Discussion

### Principal Findings

This study explored the relationships between positive aging perception, quality-of-life enhancement, social support, and various factors related to technology acceptance and readiness among older adults.

The preliminary analysis using correlation analysis revealed significant associations between demographic factors and key variables, such as age being positively related to marital status and negatively associated with quality-of-life enhancement and gerontechnology confidence; educational level being negatively correlated with living arrangements and gerontechnology confidence; and social support being positively correlated with quality-of-life enhancement. These insights suggest that the older adults who participated in this study predominantly consisted of married individuals with varying educational backgrounds, most of whom lived with household members and received substantial social support, which in turn influenced their perceptions and confidence regarding assistive technologies.

The primary analysis using structural equation modeling indicated that a positive perception of aging significantly enhances gerontechnology confidence and comfort, with a marginal positive relationship with control beliefs. These findings suggest that older adults who maintain a positive outlook on aging are more confident and comfortable with using gerontechnology, which aligns with existing literature emphasizing the role of positive aging attitudes in technology adoption [41,42]. However, quality-of-life enhancement and social support did not exhibit significant direct effects on most components of technology acceptance and readiness within the structural model. This contrasts with some previous studies that highlighted the importance of these factors in technology adoption [43,44], indicating that their influence may be more complex or mediated by other variables not captured in this model.

The post hoc analysis using MANCOVA further revealed that quality-of-life enhancement and social support significantly impact technology acceptance and readiness when controlling for demographic characteristics. Specifically, both quality-of-life enhancement and social support were found to negatively influence security perceptions, suggesting that as these factors increase, concerns about the security of assistive technologies decrease. This negative influence on security perceptions could be explained by demographic factors such as educational level and ethnicity. Educational level significantly predicted gerontechnology confidence, indicating that individuals with higher education may feel more confident in using technology, thereby reducing their security concerns. Similarly, ethnicity emerged as a significant predictor of optimism, suggesting that cultural or social backgrounds influence positive expectations toward technology, which can also alleviate security apprehensions. These findings highlight that while quality-of-life enhancements and social support do not directly influence the larger set of technology acceptance factors, they play a crucial role in alleviating security-related concerns, which are vital for the overall readiness to adopt assistive technologies.

Interestingly, social support showed negative associations with gerontechnology confidence and security perceptions in the structural model, although these were not statistically significant. This counterintuitive finding, if significant, may indicate that excessive reliance on social support could undermine individuals’ confidence in using technology independently or heighten concerns about privacy and security. Such dynamics, which contrast past observations [45-48], warrant further investigation to understand the complex role of social support in technology adoption among older adults. One possible explanation is the influence of social norms, where older adults may experience expectations or pressures from their social networks that discourage full technological engagement [49]. An overreliance on assistance from others might also reduce the perceived need for technology, leading to decreased confidence and increased security concerns. Privacy and trust issues related to technology could also contribute to these negative relationships, as older adults may fear privacy breaches or distrust the reliability of assistive technologies [50,51].

The significant influence of demographic characteristics, particularly educational level and ethnicity, emphasizes the importance of considering sociodemographic factors in technology acceptance models. Higher educational levels were associated with greater confidence in using gerontechnology, likely due to better technological literacy and problem-solving skills acquired through education. Ethnic background influencing optimism suggests that cultural factors play a role in shaping positive attitudes toward technology adoption. These findings extend previous research [43,52] by quantifying the impact of these demographic factors and highlighting their specific effects on different aspects of technology acceptance.

Contrary to initial expectations, this study did not find significant associations between positive aging perception and several factors, including attitudinal beliefs, behavioral intention, innovativeness, optimism, and security, whereas quality-of-life enhancement and social support did not produce significant relationships. This may suggest that older adults’ attitudes toward indoor assistive technologies may be more complex or mediated by other factors such as perceived need, technology complexity, or previous experience with similar devices that were not considered in this study [19,49]. While the lack of significant associations may seem surprising, it is important to acknowledge that the adoption and acceptance of assistive technologies among older adults are influenced by multifaceted factors [53]. The variables examined herein might be influenced by additional variables or interact with each other in ways that were not captured in this particular study. Future studies could explore other variables or contextual factors, such as cognitive abilities, physical health, technological literacy, or specific characteristics of the assistive technologies themselves, to develop more comprehensive models of technology acceptance among older adults [54].

### Implications and Recommendations

This study underscores the importance of fostering positive aging perceptions to enhance gerontechnology confidence and comfort among older adults. To achieve this, stakeholders should develop and implement educational programs and workshops that clearly demonstrate the benefits and practical applications of assistive technologies. These initiatives can help older adults build a more optimistic outlook on aging and increase their confidence in using these devices effectively.

Integrating quality-of-life enhancements and social support into the design and implementation of assistive technologies is crucial for addressing security concerns. Assistive devices should incorporate features that directly improve users’ daily living experiences, such as intuitive user interfaces and robust safety mechanisms. These enhancements can help reduce apprehensions about the reliability and security of the technologies, making them more acceptable and trustworthy for older adults.

Demographic factors, including educational level and ethnicity, significantly influence technology confidence and optimism. To accommodate varying educational backgrounds, assistive technology solutions should offer clear instructions, accessible interfaces, and comprehensive training materials. Technologies should also be culturally tailored to respect and integrate the diverse cultural preferences of different ethnic groups. This cultural sensitivity can promote greater acceptance and positive attitudes toward the use of assistive technologies among ethnically diverse populations.

The negative associations observed between social support and gerontechnology confidence and security perceptions indicate that excessive reliance on social networks might undermine independent technology use and heighten security concerns. To address this, support systems should be designed to empower older adults to use assistive technologies independently. Training programs for caregivers and family members should focus on encouraging autonomous use of technology rather than providing constant assistance. Addressing privacy and trust issues through transparent data handling practices and robust security features should further help alleviate security concerns, thereby enhancing older adults’ readiness to adopt assistive technologies.

To this end, enhancing technology acceptance and readiness among older adults requires a comprehensive approach that includes promoting positive aging attitudes, improving quality of life through targeted technology features, tailoring solutions to meet diverse educational and cultural backgrounds, and structuring social support to foster independence. Implementing these strategies should facilitate the effective adoption of indoor assistive technologies, ultimately improving the independence and well-being of older adults.

### Limitations and Future Directions

This study presents several limitations that should be considered when interpreting the findings. First, the sample comprised older adults aged 60 years and older who participated in an indoor setting, predominantly Chinese and married, with most living with household members. While this demographic reflects typical patterns in urban Malaysian environments, it may limit the generalizability of the results to wider populations, including those in rural areas or living in assisted living facilities. Future research should use stratified sampling techniques to include a more diverse range of settings and demographics, enhancing the applicability of the findings across different contexts.

Second, the cross-sectional design of the study restricts the ability to establish causal relationships between variables. The associations identified provide a snapshot at a specific point in time but do not account for changes and developments over time. Longitudinal studies or experimental designs would be beneficial in examining the causal effects and temporal dynamics between positive aging perception, quality-of-life enhancement, social support, and technology acceptance and readiness.

Third, reliance on self-report measures to assess constructs such as positive aging perception, quality-of-life enhancement, social support, and technology acceptance introduces potential biases, including social desirability and recall bias. These biases may affect the accuracy of the data collected. Future studies could incorporate objective measures or combine self-report instruments with other assessment methods to achieve a more comprehensive understanding of these constructs.

Fourth, this study focused on specific variables—positive aging perception, quality-of-life enhancement, and social support—to explore their influence on technology acceptance and readiness. Other relevant factors, such as cognitive abilities, physical health, technological literacy, and specific characteristics of assistive technologies, were not included in the analysis. Incorporating a wider range of variables in future research would provide a more comprehensive view of the factors influencing older adults’ technology acceptance and readiness. Using a multidimensional framework could capture the complex interactions among various factors more effectively.

Finally, the sample primarily consisted of Malaysian participants living in urban areas, which limits the diversity and generalizability of the findings to other cultural or demographic groups. Future studies should endeavor to include a more varied sample to examine potential cultural or contextual differences in the relationships between the variables. Using cross-cultural research designs or multisite studies can help identify and account for cultural and contextual variations, thereby enhancing the relevance and applicability of the findings across different populations.

### Conclusion

Understanding the factors that influence older adults’ acceptance and readiness to adopt indoor assistive technologies is essential for enhancing their independence and well-being. This study demonstrates that a positive perception of aging significantly increases gerontechnology confidence and comfort among older adults, while quality-of-life enhancements and social support play crucial roles in reducing security concerns related to technology use. Demographic factors, particularly educational level and ethnicity, also significantly influence confidence and optimism toward technology adoption. These insights highlight the need for targeted strategies to effectively promote the adoption of assistive technologies. Future research should explore the underlying mechanisms that drive these relationships and develop customized interventions to support successful technology integration among older adults.

## Abbreviations

AARC: Awareness of Age-Related Change
CFA: confirmatory factor analysis
CFI: comparative fit index
GFI: goodness-of-fit index
IFI: incremental fit index
MANCOVA: multivariate analysis of covariance
PGFI: parsimony goodness-of-fit index
RMSEA: root mean square error of approximation
TLI: Tucker-Lewis index
TPB: Theory of Planned Behavior
TRQ: Technology Readiness Questionnaire
WHO: World Health Organization
